# Artificial Neuron Based on Electrical Anisotropy from WSe_2_ Field Effect Transistors

**DOI:** 10.1002/advs.202515893

**Published:** 2026-01-20

**Authors:** Qi Sun, Ping Chen, Kun Lv, Chuanwen Chen, Jinsheng Zhu, Zhiling Chen, Yaxian Lu, Ni Zhang, Zongqian Tan, Tao Lin, Caofeng Pan

**Affiliations:** ^1^ Center on Nanoenergy Research Guangxi Key Laboratory For Relativistic Astrophysics School of Physical Science and Technology Guangxi University Nanning China; ^2^ Institute of Atomic Manufacturing, Beihang University Beijing China

**Keywords:** artificial dendrite, artificial neuron, axon‐multisynapse, electrical anisotropy, layered WSe_2_

## Abstract

Neuromorphic systems have been recognized as potential computational platforms for overcoming the von Neumann architecture, where neurons are the basic units for neuromorphic computing. However, artificial neurons of two‐dimensional materials suffered from limited stability and complex architecture. In this work, a multi‐terminal neural device was constructed based on the electrical anisotropic properties of WSe_2_. Axon‐multisynaptic performance with modulating plasticity was realized successfully by a stable six‐terminal WSe_2_ field effect transistor. And dendritic functionality with optoelectronic synergy was obtained, showcasing the functional integrity of this neural device. Recognition accuracy of handwritten digits was obtained to 97% based on the synaptic weight of an artificial neuron. Additionally, gesture control of a robot manipulator was achieved with anisotropic synaptic responses from a WSe_2_ sample. These findings not only provide novel material platforms and circuit design solutions for constructing artificial neural networks but also mark a significant advancement in the development of neuromorphic electronics capable of processing complex signals.

## Graphical abstract

An artificial neuron was realized based on the anisotropic mobility of WSe_2_. Dendritic and axon‐multisynaptic performance of a neuron was achieved with optical and voltage pulses. Recognition accuracy of handwritten digits was obtained to 97% based on the synaptic weight of an artificial neuron. And gesture control of a robot manipulator was succeeded with anisotropic synaptic responses from a WSe_2_ sample.

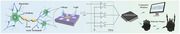

## 1 Introduction

Neuromorphic computing, as a model inspired by the human brain, provides a more flexible and efficient way to process information for the explosive growth in data‐eccentric applications [1]. This emerging technology, capable of batch parallel computation, is expected to overcome the fundamental limitations of current von Neumann computing architectures, where computing and storage are separated [2, 3, 4]. Among the developed strategies, memristor and memory transistor arrays based on 2D materials facilitate the creation of high‐performance artificial synapses with ultra energy‐efficiency [5], thanks to the unique properties of 2D materials (e.g. hierarchical structure [6], mechanical flexibility [7], and the ability to be stacked to form heterojunctions [8]), as well as the excellent performance of the devices [5, 6, 7, 8, 9, 10]. In recent years, significant efforts have been devoted to the design and fabrication of artificial synaptic devices of 2D materials [11, 12, 13, 14, 15, 16, 17, 18, 19, 20, 21, 22, 23, 24, 25, 26, 27, 28]. However, the growing demand for functional diversity and integration in neuromorphic systems underscores the urgent need to develop artificial neurons.

**FIGURE 1:**
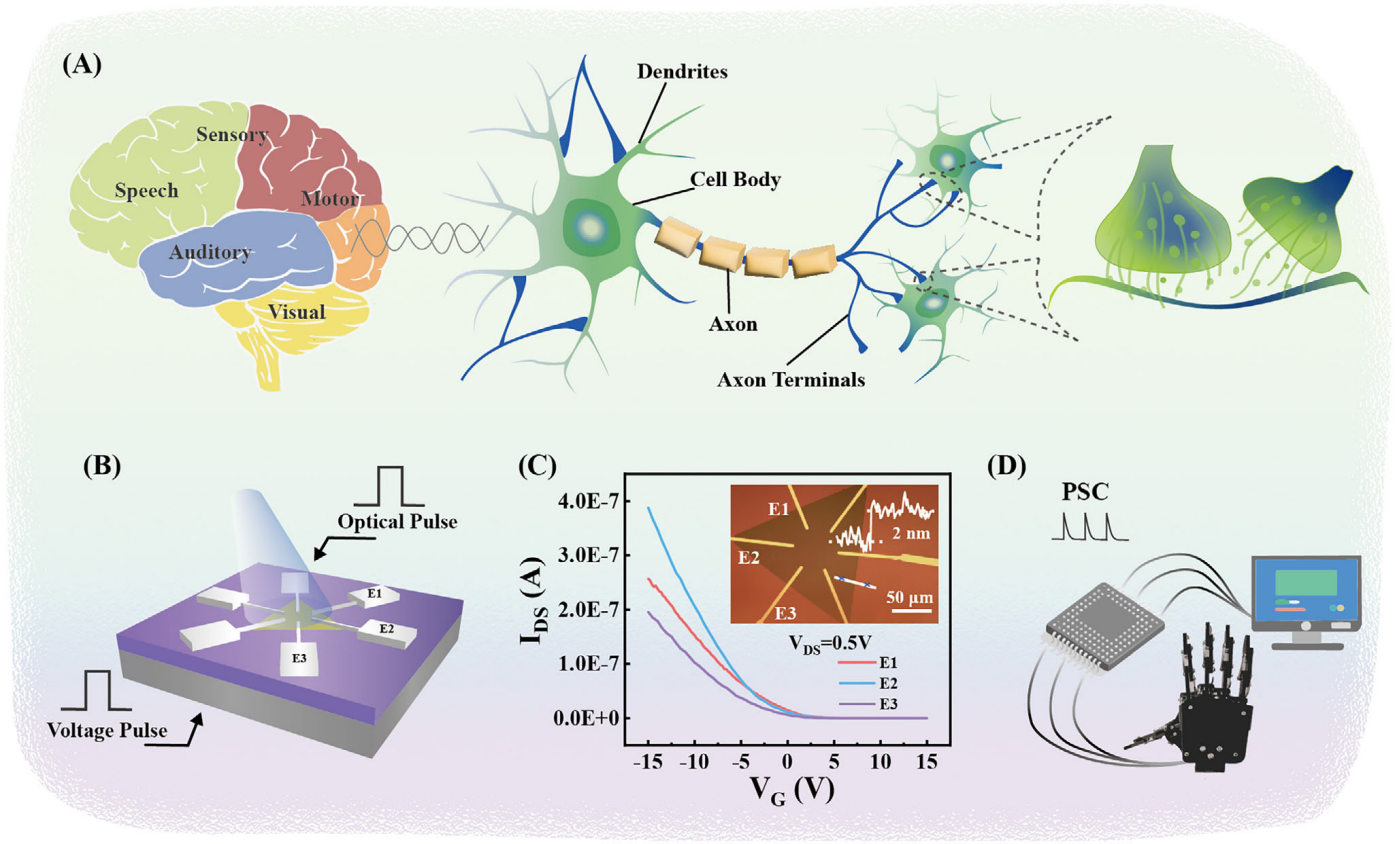
(A) Schematic of human brain functional areas and the components of neurons. (B) Structure schematic of axon‐multisynaptic neural components based on WSe_2_ FETs. (C) Transfer curves for angle‐resolved diagonal electrodes of WSe_2_ FETs. The inset shows the optical microscope image and AFM height of the WSe_2_ sample. (D) Schematic of controlled fingers of a robot manipulator by WSe_2_ FETs.

Neurons are composed of dendrites, the cell body, and the axon [29], where dendrites are responsible for receiving signals from other neurons, the cell body serves as a processing incoming signals and generating replies, and the axon transmits information to the next neurons (Figure 1A). The basic performance of the neuron is that multiple signals are received and processed in parallel. For artificial neurons, the optoelectronic anisotropy of 2D materials plays an excellent role in axon‐multi‐synaptic structures [30, 31, 32, 33, 34, 35, 36, 37, 38, 39, 40, 41, 42, 43, 44, 45, 46, 47]. This architecture allows neurons to transmit information to multiple targets simultaneously, facilitating integration and coordination to enhance processing efficiency, adaptability, and learning capabilities.

Several categories of 2D materials have been applied in neuromorphic systems. Inherent anisotropy in the material's crystal structure and band structure was first explored [30, 31, 32, 33]. For instance, Tian et al. mimicked the heterogeneity of biological synapses to realize an axon‐multisynaptic network based on anisotropic 2D black phosphorus [30]. Qin et al. exploited the intrinsic low symmetry of trigonal selenium (t‐Se) with a heterogeneity ratio as high as 8.6 and achieved a heterogeneous axon‐multisynaptic network based on different signal processing along its *c*‐axis and *a*‐axis [31]. However, those materials with intrinsic anisotropy are sensitive to oxygen, which adversely affects the stability of the devices. Then, defect engineering was developed to modulate optoelectronic anisotropy by controlling conduction in various directions by introducing specific defects, such as lattice defects, impurity atoms, or intrinsic defects [34, 35, 36, 37, 38, 39, 40]. These defects could create localized electronic states and modify carriers’ mobility and recombination dynamics, thereby influencing the 2D material's optoelectronic behavior. Vinod K. et al. demonstrated the heterosynaptic functionality based on six‐terminal MoS_2_ transistors by bias‐induced defect motion in polycrystalline MoS_2_ [36]. Subsequently, Liu et al. utilized anisotropic synaptic characteristics in initially isotropic MoS_2_ devices by localized electron beam irradiation (EBI) [38]. And yet the introduction of defects makes it challenging to achieve consistent performance because of the fluctuations of material properties [40]. The control and optimization of defect types and quantity remain a complex and unresolved challenge in defect engineering. Recently, anisotropy was achieved from microstructural engineering. Heterostructures composed of stacked 2D materials bound exhibited interlayer electronic interactions and induced anisotropy in energy band alignment, carrier mobility, and optical properties across different layers [41, 42, 43, 44]. Besides, device architecture design also played a critical role in modulating optoelectronic performance [45, 46, 47]. Chen et al. developed a wrinkled rhenium disulfide (ReS_2_) transistor, where the wrinkled morphology enhanced carrier trapping and de‐trapping at dielectric interfaces. This design enabled the generation of three distinct datasets corresponding to three orientations, suffering from the separated devices and the limitation of practical integration. [45]

Herein, we developed a neural device with a simple preparation process based on the stability and electrical anisotropy of WSe_2_ [48], where three pairs of diagonal electrodes were constructed to simulate an axon‐multisynaptic structure. Optical and electronic signals are heterogeneously transmitted to the WSe_2_ field effect transistor (FET) by synaptic weight modulation, enabling selective responses of neurons to external stimuli. In other words, artificial dendrite responses to optics and electronics. Then, the electrical anisotropy of WSe_2_, originating from anisotropic mobility induced by the intrinsic shielding layer, enables different outputs in various crystal directions to simulate the axon performance. Thus, multiple signals of inputs and outputs have been achieved in one WSe_2_ sample to mimic the biological neurons. Additionally, precise manipulation of robotic fingers was achieved through anisotropic synaptic responses from multiple electrode pairs in FETs (Figure 1D).

This work establishes a flexible neuromorphic platform for efficient information processing, particularly in complex multimodal sensory tasks.

## 2 Results and Discussion

### 2.1 Constructing an Artificial Neuron Based on Electrical Anisotropy

WSe_2_ was chosen for artificial neurons due to its excellent optoelectronic properties. Layered WSe_2_ was grown on a silicon substrate with an oxide layer using the physical vapor deposition (PVD) method (Figure S1) [49]. Raman spectra were measured to confirm the crystal structure (Figure S2A). The peaks at 250 and 260 cm^−1^ corresponded to the E^1^_2g_ and A_1g_ modes of 2H WSe_2_, respectively. The peak at 309 cm^−1^ (B^2^_g1_) originated from interlayer interactions, agreeing well with a previous report [50]. The atomic force microscopy (AFM) image (Figure S2B) shows the thickness of the material to be 2 nm. Three pairs of Ag diagonal electrodes (E1, E2, and E3) were designed on WSe_2_ spaced at an angle of 60° (Figure 1B; Figure S3) to construct a six‐terminal neural component. Subsequently, transfer curves were tested to evaluate electrical properties (Figure 1C) with *V_DS_* at 0.5 V. The *I_DS_* of WSe_2_ FET enhanced with the negative increase of the gate voltage, indicating the P‐type channel behavior of WSe_2_ with a high on/off current ratio (>10^6^). At the same time, it is clearly found that the *I_DS_* increased first from E1 to E2, then decreased from E2 to E3, suggesting anisotropic carrier transport in different directions.

In order to understand electrical anisotropy in WSe_2_ FET devices, contacts between the electrodes and the WSe_2_ were considered. The non‐linear output curves show the Schottky contact between WSe_2_ and Ag electrodes (Figure S4). The Schottky barrier height (SBH) was calculated with temperature‐dependent output curves (Figure S5). The SBH values for the E1, E2, and E3 electrode pairs were calculated to be 85.4±10.9 meV, 74.9±5.37 meV, and 99.2±1.67 meV, respectively. The different SBH will induce the changed current from exp(‐4.63012E‐19) to exp(‐6.12819E‐19) according to *I_DS_* ∝exp[‐*qΦ_B_/k_B_T*] [51]. The little current difference caused by SBH suggests not the main origin of electrical anisotropy from six‐terminal WSe_2_ FETs. Thus, the electrical transmission in the WSe_2_ channel should be paid attention. Intrinsic shielding layer modulated anisotropic carrier mobility in 2D WSe_2_ was proposed and confirmed in theoretical and experimental results, where the cluster and adlayer of Se and W atoms (Figure S6) could induce defect scattering to reduce conductivity and make WSe_2_ become metallic to enhance the electrical conductivity, respectively [48]. Thus, the electrical anisotropy of carrier mobility spans from 0.16 to 0.95 for WSe_2_ FET at a gate voltage of −60 V. Thereby, the intrinsic shielding layer might be the reason for anisotropic carrier mobility in 2D WSe_2_ FETs, which provides the possibility to simulate the axon of artificial neurons.

### 2.2 Electrical Dendrite Simulation

**FIGURE 2:**
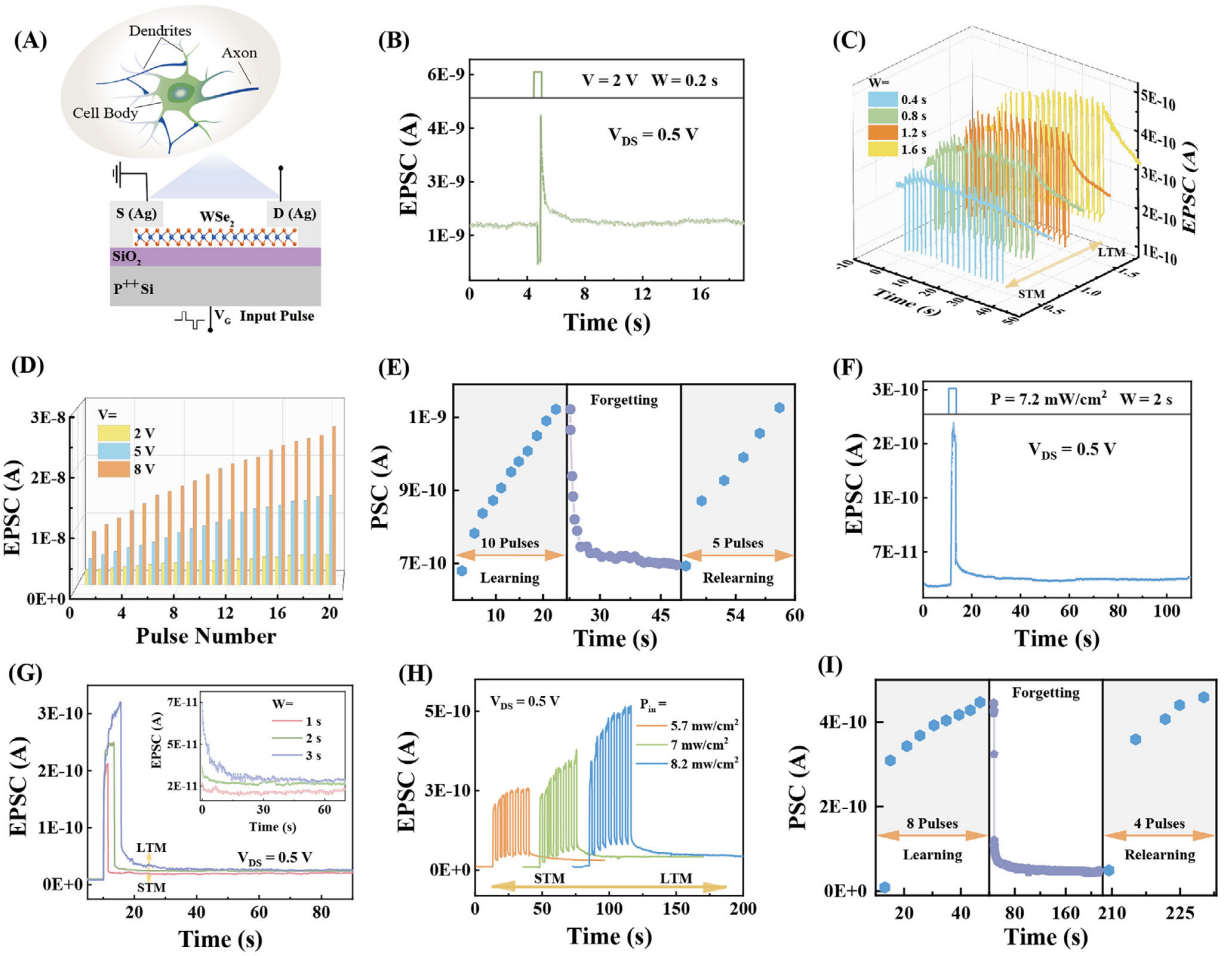
Optoelectric dendrite simulation. (A) Schematic diagram of dendritic structure and WSe_2_ FET device. (B) EPSC is excited by a voltage pulse. (C) Modulated EPSC triggered by different voltage pulse widths. (D) EPSC induced by different amplitudes. (E) The “learning—forgetting—relearning” processes were simulated by voltage pulses. (F) EPSC stimulated by an optical pulse. (G) Modulated EPSC by varying the width of the optical pulse. (H) Modulated EPSC by varying the optical intensity. (I) The “learning—forgetting—relearning” processes were simulated by optical pulses.

For biological neurons, one of the important performances is that multiple signals can be received and handled by the dendrite and cell body. The optoelectronic dendrite simulation based on laminar WSe_2_ was initially implemented (Figure 2A). In this configuration, the channel conductance of WSe_2_ corresponding to synaptic weights and synaptic plasticity can be modulated through both optical and electrical signals.

The transfer characteristics of the device exhibited hysteresis behaviors when gate voltage was applied in the loop of different gate voltages (Figure S7), suggesting an essential feature of plasticity modulation and memory window by an electrical signal [52]. The hysteresis in the transfer characteristics did not show a sudden transition between high resistance and low resistance, indicating ion‐migration driven conductive filament switching in oxide‐based memristors didn't response to it [53]. The mechanism is attributed to charge trapping and release at the interface between the dielectric layer and the channel [54, 55]. Additionally, when increasing the back‐gate voltages from ±2 to ±10 V, the hysteresis behaviors progressively enlarged, demonstrating widened memory windows. This effective regulation of gate voltages underscores the device's potential for applications in neural networks.

Synaptic plasticity was simulated by polarity, width, and amplitude of gate pulse voltages (Figure 2B–D). First, a positive voltage pulse with an amplitude (*V*) of 2 V, a pulse interval (*Δt*) of 0.5 s, and a pulse width (*W*) of 0.5 s was applied to the device (Figure 2B). The current in the P‐type WSe_2_ channel sharply decreased because electrons were injected into the channel (Figure S8A(i)), accompanied by trapping some of the injected electrons in the interface between WSe_2_ and SiO_2_ (Figure S8A(ii)). After canceling the positive pulse, the electrons in the WSe_2_ channel were released, resulting in an increased hole concentration and higher post‐synaptic current [54, 55]. Subsequently, the post‐synaptic current was decayed slowly to the initial state for the gradual release of trapped electrons in Figure S8A(ii). The behavior is consistent with the characteristics of excitatory post‐synaptic current (EPSC) in biological neural systems. Conversely, when a negative voltage pulse was applied (Figure S8A(iii) and (iv)), holes were injected into the WSe_2_ channel, causing a sharp increase in *I_DS_* (Figure S9). As trapped electrons in defects were gradually released and recombined with holes, the channel current decreased [54, 55], aligning with the characteristics of inhibitory post‐synaptic current (IPSC). Therefore, positive pulses applied to the gate voltage induce EPSC, while negative pulses induce IPSC, agreeing well with the hysteresis curves observed in the transfer characteristics shown in Figure S7. Furthermore, synaptic weights of artificial dendrites were modulated by the width and amplitude of the voltage pulses. When increasing the pulse width from 0.4 to 1.6 s (Figure 2C), it was observed that longer pulse widths resulted in larger EPSCs and extended retention times after the pulse termination, indicating the transition from short‐term memory (STM) to long‐term memory (LTM). At the same time, when positive pulses (*W* = 0.5 s, *Δt* = 0.5 s) with various amplitudes (*V* = 2, 5, and 8 V) were applied, the EPSC exhibited a continuous increase (Figure 2D), suggesting that synaptic weights can be progressively strengthened through repeated presynaptic neuronal stimulation. This phenomenon is consistent with long‐term potentiation (LTP) in biological nervous systems, where continuous stimulation enhances synaptic transmission efficiency [56]. In the opposite way, when negative pulses (*W* = 0.5 s, *Δt* = 0.5 s) were applied (Figure S10), IPSCs were induced, mimicking the long‐term depression (LTD) phenomenon in biological neural networks, where synaptic efficacy is persistently reduced. Thus, by precisely tuning the width, amplitude, and polarity of the voltage pulses, not only can synaptic weights be accurately regulated, but also the process of synaptic strengthening under repeated stimulation can be effectively replicated. This adjustable synaptic plasticity implies the capability to emulate biological learning and memory experiences.

Learning and memory experiences were further demonstrated by learning, forgetting, and relearning (Figure 2E). In the initial learning phase, the EPSC of the device gradually increased from 7.5 × 10^−10^ A to 1.12 × 10^−9^ A with the application of 10 voltage pulses. Then, the EPSC decayed over 26 s after stopping the stimulation, mimicking the process of learning and subsequent forgetting in the human brain. During the relearning stage, when the same pulses were reapplied, the current reached 1.12 × 10^−9^ A only by the fifth pulse. During this relearning process, because the electrons captured by interface defects are not fully released after removing the first pulses, when the same pulses were reapplied, repeated trapping and accumulation of charge were promoted, which led to an increase in current and accelerated weight update [23]. This phenomenon indicates that, for the same cognitive level, fewer pulses were required to achieve the initial learning level during relearning, aligning closely with the human learning and memory experiences, where relearning is more efficient than the initial learning process.

### 2.3 Optical Dendrite Simulation

Optical signals were employed to regulate the synaptic plasticity of the artificial dendrite (Figure 2F–H). Upon irradiation with a 405 nm laser pulse (*p* = 7.2 mW/cm^2^, *W* = 2 s), the post‐synaptic current presented a sudden increase (Figure 2F), followed by an exponential decay after removing irradiation. The post‐synaptic current decayed over 95 s. The sudden increase is attributed to added electron‐hole pairs generated by irradiation, where the boosted carrier concentration enlarged the channel conductance, accompanied by lots of electrons being trapped by intrinsic and interface defects (Figure S8A(v)). And the exponential decay originates from the recombination of electrons and holes generated by irradiation after removing the laser. The gradual decay over 95 s to its pre‐illumination state corresponds to the slow release of trapped electrons (Figure S8A(vi)). The increase and exponential decay of post‐synaptic current successfully represented the modulation of synaptic plasticity via optical signals. Subsequently, optical‐dose‐dependent synaptic plasticity was investigated with changing pulse width (Figure 2G) and intensity (Figure 2H). When the pulse width was increased from 1 to 3 s (Figure 2G), the EPSC was raised from 0.21 to 0.32 nA. The longer the artificial dendrite is exposed to the illumination, the larger the EPSC amplitudes are presented, suggesting enhanced synaptic connectivity. At the same time, a prolonged relaxation time was observed with increasing pulse width, effectively simulating the transition from STM to LTM. Besides, the synaptic strength of the artificial dendrite could be modulated by varying the optical intensity (Figure 2H). The increasing intensities of optical pulses led to a corresponding rise of ΔEPSC, further replicating the transition from STM to LTM. All of the results highlight the capability of emulating synaptic plasticity and memory processes through optical signals. The optical signal‐based learning experiences were also confirmed (Figure 2I). During the initial application of an optical pulse sequence (*p* = 7.2 mW/cm^2^, *W* = 4 s, *Δt* = 1 s), the EPSC of the device gradually increased from 3.6 × 10^−10^ A to 4.5 × 10^−10^ A after 8 optical pulses. Then, EPSC decayed over 163 s when the optical signals ceased. Upon illumination by the same pulse sequence, the EPSC reached 4.6 × 10^−10^ A only by the fourth pulse, demonstrating accelerated relearning. Finally, the application of optical and voltage pulses jointly modulated LTP and LTD (Figure S12A), indicating that synaptic plasticity in artificial dendrites can also be achieved through combined optical and electrical stimulation.

### 2.4 Plasticity of Artificial Axon Modulated by Electrical Signals

**FIGURE 3:**
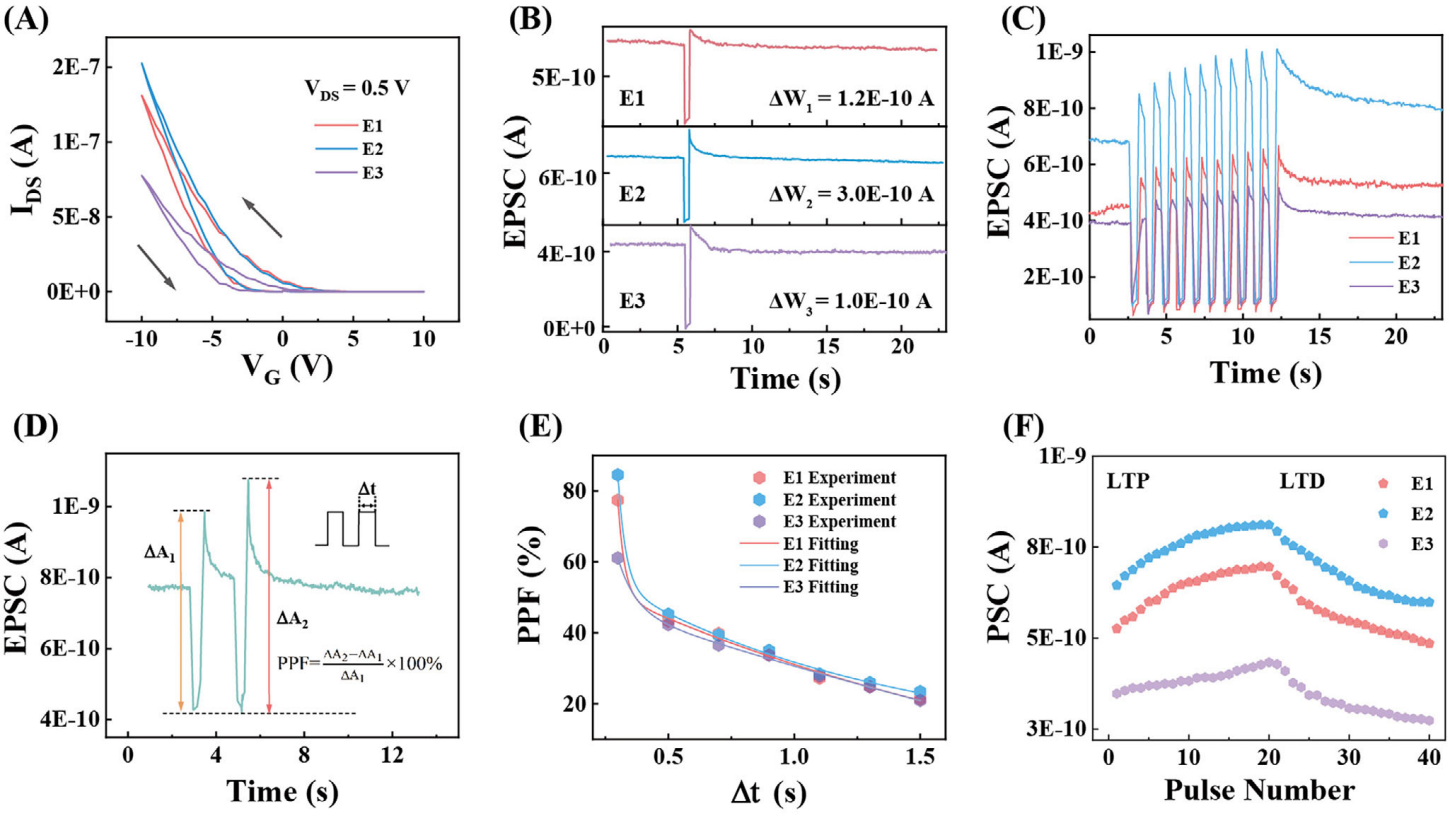
Artificial axon plasticity modulated by electrical signals. (A) Transfer hysteresis characteristic curves of the six‐terminal artificial axon. (B) EPSC of the six‐terminal artificial axon induced by the same positive pulse (*V* = 1.5 V, *W* = 0.5 s). (C) Multi‐stage conductance state under a continuous voltage pulse (*V* = 2 V, W = 0.5 s, *Δt* = 0.5 s, *N* = 10). (D) EPSC excited by a pair of voltage pulses (*V* = 2 V, *W* = 0.5 s, *Δt* = 1.3 s). (E) Dependence of PPF index on pulse interval *Δt*. (F) LTP and LTD behaviors of a six‐terminal artificial axon (*V* = ±1.5 V, *W* = 0.5 s, *Δt* = 0.5 s) with *V*_*DS*_ at 0.5 V.

Another essential function of biological neurons is transmitting information to the next several neurons simultaneously, which is carried out by the axon (Figure 1A). Thus, the artificial neuron with a six‐terminal axon structure was designed to achieve this function (Figure 1B). The transfer hysteresis curves of the six‐terminal axon structure were measured first (Figure 3A) with *V_DS_* at 0.5 V. The hysteresis and significant electrical anisotropy of transfer curves from E1 to E3 imply the potential application in different synaptic behaviors excited by the same stimulation. Then, the same positive voltage pulse (*V* = 1.5 V, *W* = 0.5 s) was applied to the back gate, and EPSCs were induced in the artificial six‐terminal axon structure (Figure 3B). Although all of the channels exhibited excitatory synaptic responses, the changes in synaptic weights (*ΔW*) varied significantly from E1 to E3, where *ΔW*s for E1, E2, and E3 were 1.2 × 10^−10^ A, 3.0 × 10^−10^ A, and 1.0 × 10^−10^ A, respectively. Based on the results, we suggest that the anisotropic mobilities respond to varying synaptic weights from E1 to E3 (Figure S11) [48, 52]. Those various synaptic weights confirmed the basic capabilities of transmitting information to the next several neurons simultaneously. Furthermore, a pulse sequence of positive voltage (*V* = 2 V, W = 0.5 s, *Δt* = 0.5 s, *N* = 10) was applied to achieve conductance modulation. The six‐terminal axon presented various plasticity from E1 to E3 (Figure 3C), confirming diverse synaptic behaviors again.

Subsequently, the PPF index was compared to evaluate the different memory retention capacities from E1 to E3 [57]. When a pair of consecutive voltage pulses (*V* = 2 V, *W* = 0.5 s) was applied with an interval of 1.3 s, the amplitude of the second post‐synaptic current was higher than that of the first (Figure 3D), indicating the memory retention capacity and successful simulation of PPF behavior. The PPF index was introduced to quantify its dependence on the time interval and assess different memory retention capacities from E1 to E3 (Figure 3E). The PPF indices of E1, E2, and E3 are77, 84, and 61, suggesting differentiated memory retention and information processing capabilities of the six‐terminal axon structure. The different PPF indices from E1 to E3 were also suggested to the varying carrier mobilities and release of captured electrons from E1 to E3 (Figure S11). This performance is consistent with a biological axon‐multisynaptic. Besides, exponential decays of PPF indices were observed with increasing pulse interval. And the decays were fitted by a double exponential function [58]: $\mathrm{PPF}=A_{0}+A_{1}\exp(-\Delta t_{\mathrm{pre}}/\tau_{1})+A_{2}\exp(-\Delta t_{\mathrm{pre}}/\tau_{2})$, where *A*_1_ and *A*_2_ are fast and slow facilitation magnitudes, τ_1_and τ_2_are fast and slow decay times in PPF. τ_1_and τ_2_were calculated to be 26 and 2077 ms for E1, 31 and 1109 ms for E2, 71 and 5200 ms for E3, respectively. Order‐of‐magnitude difference between τ_1_ and τ_2_ from E1 to E3 is compatible with the synaptic time scale [58].

Further, the WSe_2_‐based axon‐multisynaptic neural structure was capable of LTP and LTD behaviors (Figure 3F). 20 pulse sequence of positive voltage was applied (*V* = 1.5 V, *W* = 0.5 s, *Δt* = 0.5 s), followed by 20 pulse sequence of negative voltage (*V* = ‐1.5 V, *W* = 0.5 s, *Δt* = 0.5 s). The post‐synaptic current exhibited a gradual increase during LTP and a gradual decrease during LTD, effectively replicating the excitatory and inhibitory behaviors observed in biological synaptic systems. Notably, the modulation of synaptic weights from E1 to E3 also demonstrated a strong dependence on the orientation of the channels, highlighting the ability to mimic the anisotropic nature of biological axon networks by electrical signals.

### 2.5 Optical Signals Regulated Artificial Axon Plasticity

**FIGURE 4:**
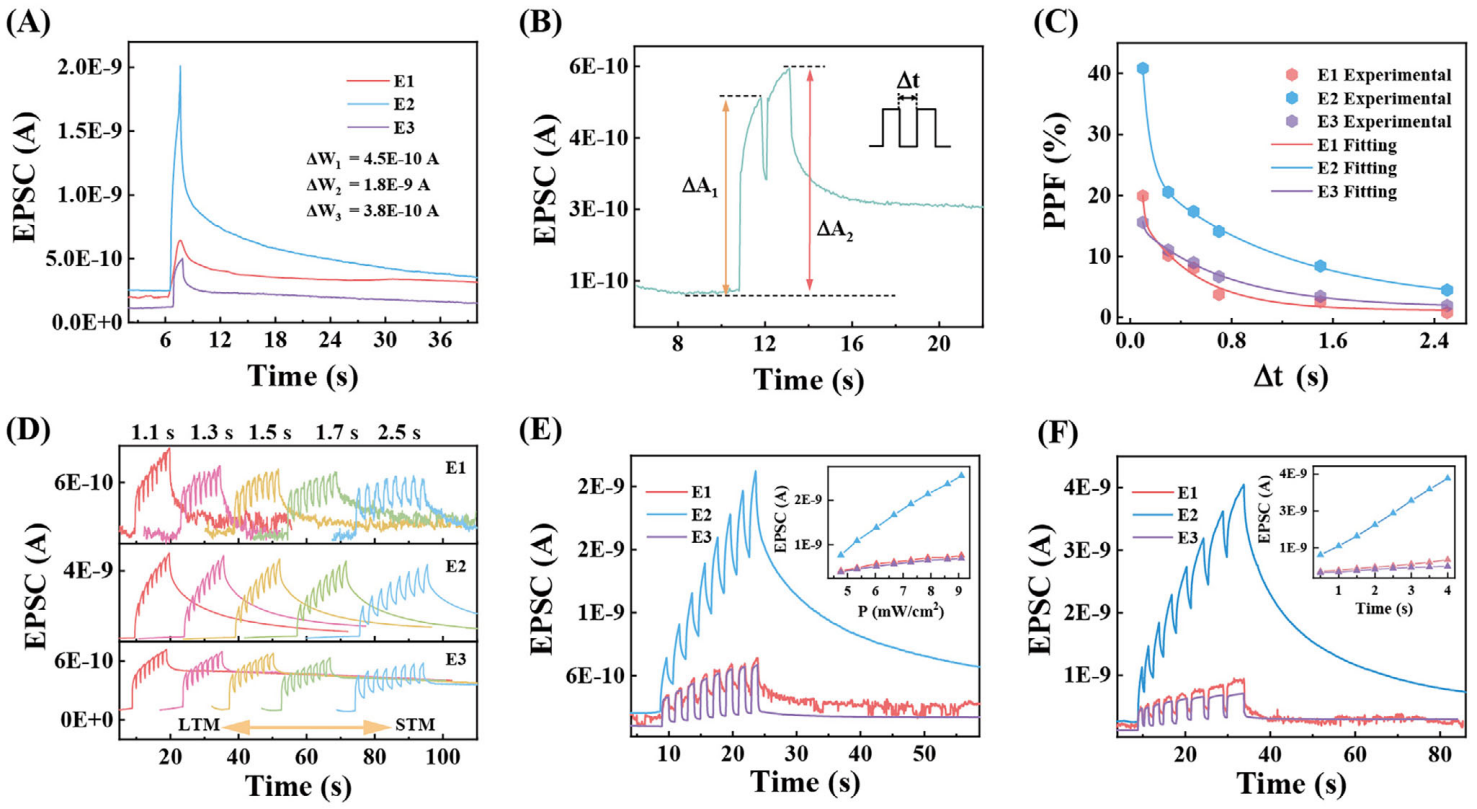
Regulated artificial axon plasticity by optical signals. (A) EPSC of the six‐terminal artificial axon induced by the same optical pulse (*p* = 6.7 mW/cm^2^, *W* = 1 s). (B) EPSC excited by a pair of optical pulses (*p* = 6.7 mW/cm^2^, *W* = 1 s, *Δt* = 0.1 s). (C) Exponential PPF indices dependence on *Δt*. (D) EPSC behaviors of the six‐terminal artificial axon triggered by different optical pulse periods. (E) EPSC triggered by optical pulses with different power (*W* = 1s, *Δt* = 1 s), inset is the dependence of the extracted EPSC of the six‐terminal artificial axon on optical intensity. (F) EPSC triggered by optical pulses with different pulse widths (*p* = 5.3 mW/cm^2^, *Δt* = 1 s), the inset shows the dependence of extracted EPSC on pulse widths. All were performed at *V_DS_* = 0.5V.

Because the artificial dendrite of the neuron could be triggered by optical signals (Figure 2F–H), the anisotropic transmission was best achieved in the six‐terminal artificial axon stimulated by optical pulses. When a single optical pulse (*p* = 6.7 mW/cm^2^, *W* = 1 s) was initially applied (Figure 4A), synaptic weights were different from each other, where *ΔW*s for E1, E2, and E3 are 4.5 × 10^−10^ A, 1.8 × 10^−9^ A, and 3.8 × 10^−10^ A, respectively. These diverse synaptic weights indicate the promising capability of differentiated communication with other neurons. Subsequently, PPF behaviors of the six‐terminal artificial axon were verified and calculated by two consecutive optical pulses (*p* = 6.7 mW/cm^2^, *W* = 1 s, *Δt* = 0.1 s) and various pulse intervals (Figure 4B,C). The various PPF indices from E1 to E3 by optical signals were in line with information transmission to all directions of the biological axons of neurons. In addition, the multilevel retention and storage states of the six‐terminal artificial axon were proved again by periods (Figure 4D), intensities (Figure 4E), and widths (Figure 4F) of optical pulses. Increasing pulse periods induced a plasticity transition from LTM to STM for the decreased memory performance, while enhanced pulse intensities and widths generated increased retention and storage states. Therefore, the six‐terminal device exhibits an anisotropic response to optical signals, agreeing well with the differential information transmission observed in biological axon‐multisynaptic structures. Besides, endurance tests with 900 consecutive pulses and stability tests under continuous power density high to 15 mW/cm^2^ for 560 s were performed, the weak attenuation of EPSC for three channels demonstrated a good reproducibility and endurance of the artificial neuron based on multi‐terminal WSe_2_ devices (Figure S13), laying a foundation for its practical applications.

### 2.6 Handwritten Digit Recognition and Gesture Control

**FIGURE 5:**
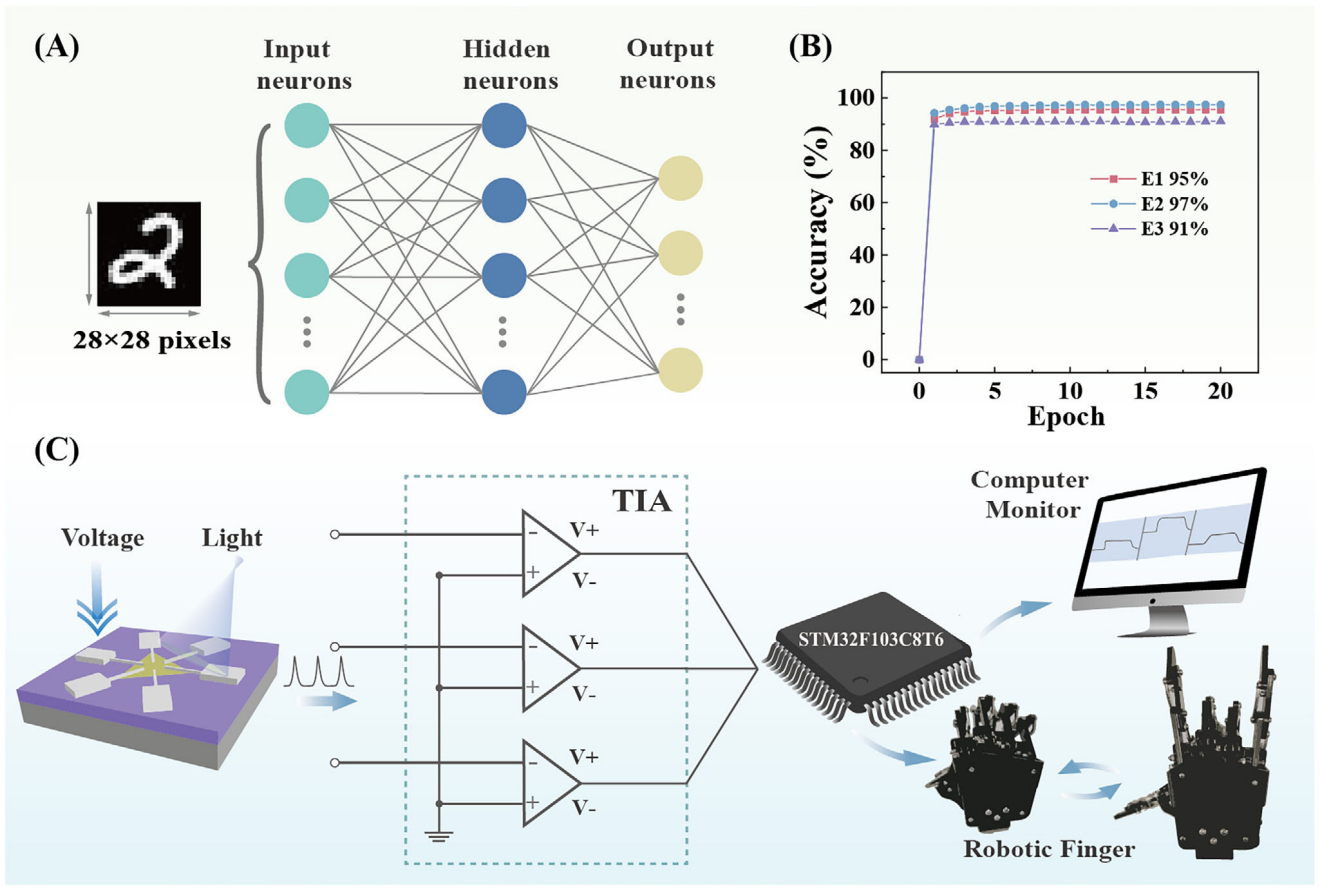
(A) MNIST dataset and neural network with the layers of 784 input neurons, 300 hidden neurons, and 10 output neurons. (B) Recognition accuracy from the MNIST simulation for each training. (C) Schematic diagram of the gesture control of a robotic manipulator by optical pulses.

The CrossSim crossbar simulator was employed to evaluate the performance of our artificial dendritic devices by simulating a deep neural network trained on the 28 × 28 MNIST dataset. The artificial neural network (ANN) was consisted by three layers: an input layer of 784 neurons (equivalent to the 28 × 28 pixels in the handwritten image), a hidden layer of 300 neurons, and an output layer of 10 (corresponding to numbers 0–9), as shown in Figure 5A. The synaptic layer of the ANN was implemented as a crossbar array (Figure S12B), with synaptic devices positioned at each crosspoint. All neurons were fully connected through 784 × 300 × 10 synaptic weights, characterized by the optical‐signal LTP and electrical‐signal LTD curves of the artificial neuron (Figure S12A). The recognition accuracy of artificial axonal devices was high to 95%, 97%, and 91% for E1, E2, and E3, respectively, indicating that the anisotropic WSe_2_‐based system exhibited consistently high recognition abilities across multiple crystal orientations (Figure 5B).

So far, we achieved artificial dendrites stimulated by opto‐electrical signals and artificial axons with the capability of anisotropic delivering information, mimicking the basic performance of artificial neurons on one six‐terminal WSe_2_ device. Combined with opto‐electrical simulation, complex information transmission might be possible based on the anisotropic responses of an artificial neuron (Figure 5C). When the optical pulses were applied, the anisotropic synaptic currents from three electrode pairs of the WSe_2_ device were detected with *V_DS_* at 0.5 V. The currents were converted to voltage signals by a trans‐impedance amplifier (gain = 10^10^ V/A, bandwidth = 10 kHz), followed by digitization with a microcontroller (STM32F103C8T6). Then, the digitized signals were, on one hand, transmitted to a computer through serial ports for visualization, on the other hand, connected to the steering gears of thumb, pointer, and pinky of a robot manipulator. The computer analyzes the voltages and waveforms from three electrode pairs of the WSe_2_ device, which were the basis for threshold voltages of the steering gears of thumb, point, and pinky of the robot manipulator. Finally, the optoelectronic currents, induced by optical pulses, controlled the stretching of the thumb, point, and pinky of the manipulator. And the bending of three fingers was realized by removing the optical pulses. In other words, the different gestures were achieved by controlling the threshold voltages of the steering gears, leading to stretching and bending of fingers (Movie S1; Figure 5C).

This process achieved dynamic mapping and synchronous feedback between finger motion states and real‐time waveform of anisotropic synaptic currents from of the WSe_2_ device. It established a complete physical mapping of “optoelectronic anisotropic signals to gesture control,” demonstrating that the device's direction‐dependent synaptic responses of the WSe_2_ device could be directly translated into controlled capabilities for neuromorphic hardware. Compared to traditional multi‐channel control relying on software algorithms, our approach achieved signal differentiation by intrinsic hardware anisotropy, reducing computational overhead and providing a more direct physical embodiment for spatial task processing in neuromorphic systems. Furthermore, a multi‑level memristive network was proposed based on multi‐terminal optoelectronic WSe_2_ devices (Figure S14). Due to the retention characteristics of the WSe_2_ devices, the written conductance states would be accumulated at the corresponding nodes and propagated along the network to the next level, endowing the whole system with history‐dependent temporal weighting behavior.

## 3 Conclusion

Layered WSe_2_ was employed to mimic an artificial neuron based on the opto‐electrical properties and electrical anisotropy, where the intrinsic shielding layers of W and Se atoms induced anisotropic carrier transmission. The artificial dendrite of the neuron was achieved by optical and electrical signals and exhibited a superior optical memory performance with an optical decay of over 95 s for a single pulse. The synaptic plasticity and memory processes, including LTP, LTD, STM, and LTM, were regulated by polarity, width, amplitude of gate voltage pulses, and intensity, period, and width of optical pulses. At the same time, an artificial axon of a neuron was fabricated and realized with six‐terminal WSe_2_ FETs. The dependence of synaptic weights and PPF indices on the directions of the WSe_2_ channel was modulated successfully by optical and voltage pulses, respectively. That diverse synaptic plasticity granted the differentiated memory retention and information processing capability of the artificial axon. Furthermore, handwritten digit recognition was achieved with the accuracy of 95% (E1), 97% (E2), and 91% (E3) based on synaptic weights of artificial axon. Finally, the robotic gesture of the manipulator was controlled via anisotropic outputs from artificial neurons. Our results not only provide a novel material platform and circuit design strategy for constructing simulated neural networks but also lay the groundwork for the development of future intelligent robotic systems capable of autonomous decision‐making in complex environments.

## 4 Experimental Section

### 4.1 Materials Synthesis

Layered WSe_2_ was grown by PVD method, as shown in Figure S1. WSe_2_ powder (99.9%, Alfa) was used as the raw material. 2 g of WSe_2_ powder was supported by a quartz boat and placed in a 1‐inch quartz tube at the center of the furnace. A Si substrate with 285 nm SiO_2_ was placed at the downstream end of the furnace. The tube was heated to 1185°C for 3 min under ambient pressure and an argon flow of 40 sccm, followed by cooling down naturally.

### 4.2 Device Fabrication

The six‐terminal FETs were fabricated by laser direct writing (Micro Writer ML3) and electron beam evaporation (PVD‐75) (in Figure S2). The positive photoresist (MICROPOSIT S1805 G2) was spin‐coated on layered WSe_2_ supported on the 285 nm SiO_2_/Si substrate at 3000 rpm for 50 s, and dried at 110°C for 2 min. Laser direct writing was employed to open the six‐terminal patterns. 50 nm silver electrodes were deposited by electron beam evaporation (PVD‐75). The sample was immersed in acetone to remove the residual positive photoresist.

### 4.3 Characterization

The morphology and thickness of WSe_2_ were studied by optical microscopy (Olympus BX43F with CCD camera) and an atomic force microscopy system (Dimension Icon). Raman spectrum was measured by a micro confocal‐based Raman spectrometer (LABRAM HR EVO), excited by 405 nm at room temperature.

### 4.4 Measurements

The electrical characterization was performed using a semiconductor parameter analyzer (Keithley 4200A‐SCS) equipped with a probe station (Lake Shore CRX‐VF). In the gesture control of the robotic manipulator, anisotropic synaptic current was converted into voltage signals by the transimpedance amplifiers (gain = 10^10^ V/A, bandwidth = 10 kHz) and digitized via a microcontroller (STM32F103C8T6).

## Conflicts of Interest

The authors declare no conflict of interest.

## Supporting information




**Supporting File**: advs73869‐sup‐0001‐SuppMat.docx.

**Supporting File**: advs73869‐sup‐0002‐MovieS1.mp4.

## Data Availability

The data that support the findings of this study are available from the corresponding author upon reasonable request.
