# Supplementary material for: Artificial Neuron Based on Electrical Anisotropy from WSe2 Field Effect Transistors
Source: Adv Sci (Weinh). 2026 Jan 20;13(17):e15893. doi: 10.1002/advs.202515893 (PMC13042943; doi:10.1002/advs.202515893)
Supplement: Supplementary file 1 — Supporting File: advs73869‐sup‐0001‐SuppMat.docx. [file ADVS-13-e15893-s002.docx]

**Supporting Information**

**Artificial neuron based on electrical anisotropy from WSe_2_ field effect transistors**

*Qi Sun^#^, Ping Chen^#,^ *, Kun Lv,* *Chuanwen Chen,* *Jinsheng Zhu,* *Zhiling Chen, Yaxian Lu, Ni Zhang,* *Zongqian Tan, Tao Lin, Caofeng Pan**

Q. Sun, Dr. P. Chen, K. Lv, C. Chen, J.Zhu, Z. Chen, Y. Lu, N. Zhang, Z. Tan, Dr. T. Lin

Center on Nanoenergy Research

Guangxi Key Laboratory for Relativistic Astrophysics

School of Physical Science and Technology

Guangxi University, Nanning, 530004, China

E-mail: [chenping@gxu.edu.cn](mailto:chenping@gxu.edu.cn) (P. Chen)

Prof. C. F. Pan

Institute of Atomic Manufacturing

Beihang University, Beijing, 100191, China

E-mail: [pancaofeng@buaa.edu.cn](mailto:pancaofeng@buaa.edu.cn) (C. F. Pan)

# Q. Sun and P. Chen have contributed equally to this work.

*Correspondence and requests for materials should be addressed to C. F. Pan ([pancaofeng@buaa.edu.cn](mailto:pancaofeng@buaa.edu.cn)) or to P. Chen ([chenping@gxu.edu.cn](mailto:chenping@gxu.edu.cn))


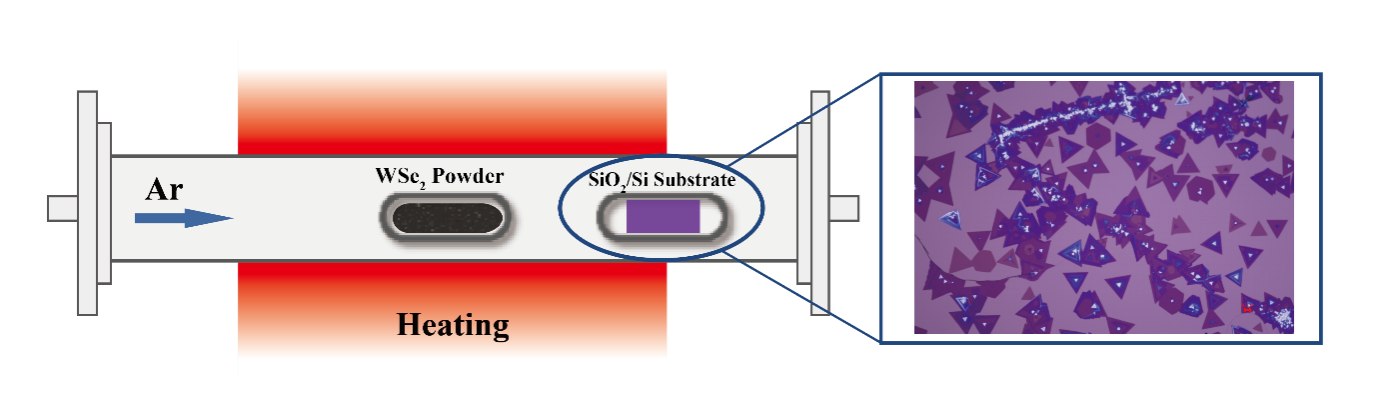


**Figure S1**. Schematic of WSe_2_ growth system by PVD method.


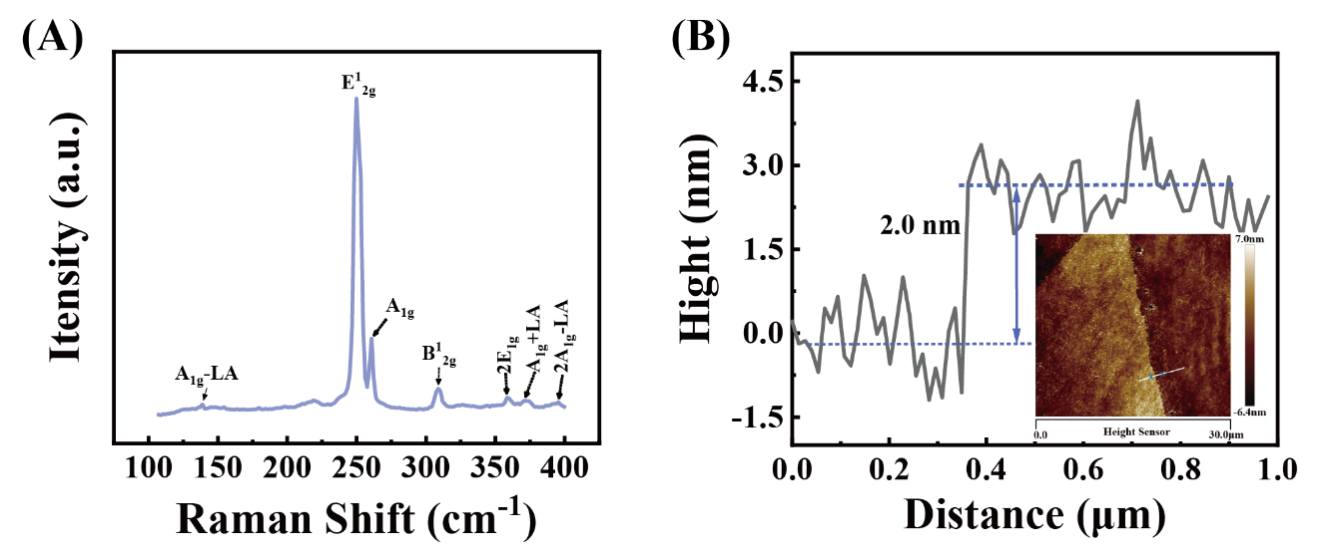


**Figure S2.** (A) Raman spectrum of WSe_2_ devices. (B) Height profile of WSe_2_ along the horizontal line in the AFM image (inset).


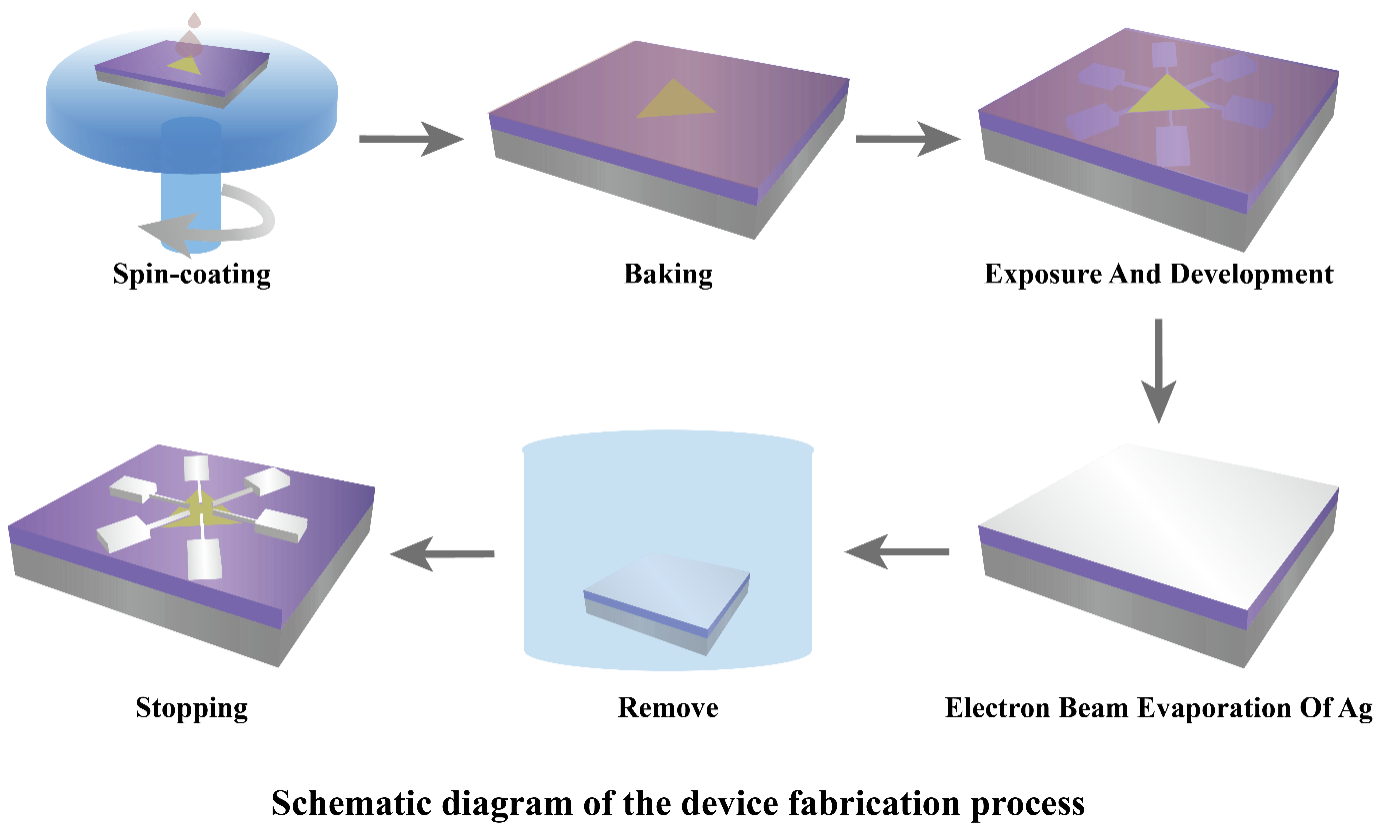


**Figure S3.** Schematic diagram of the FET fabrication process.


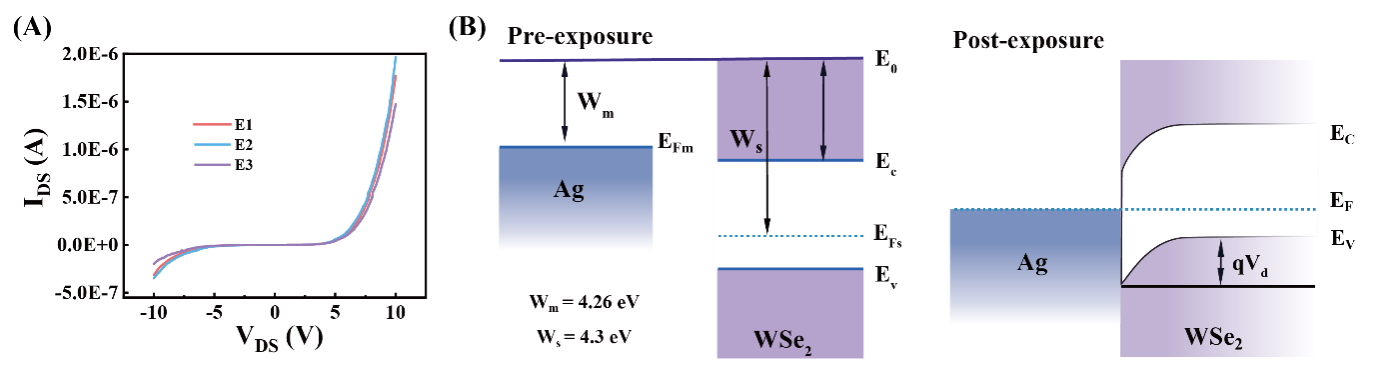


**Figure S4.** (A) Output curves of six-terminal FET devices. (B) Schematic of Schottky contact between WSe_2_ and Ag electrodes.


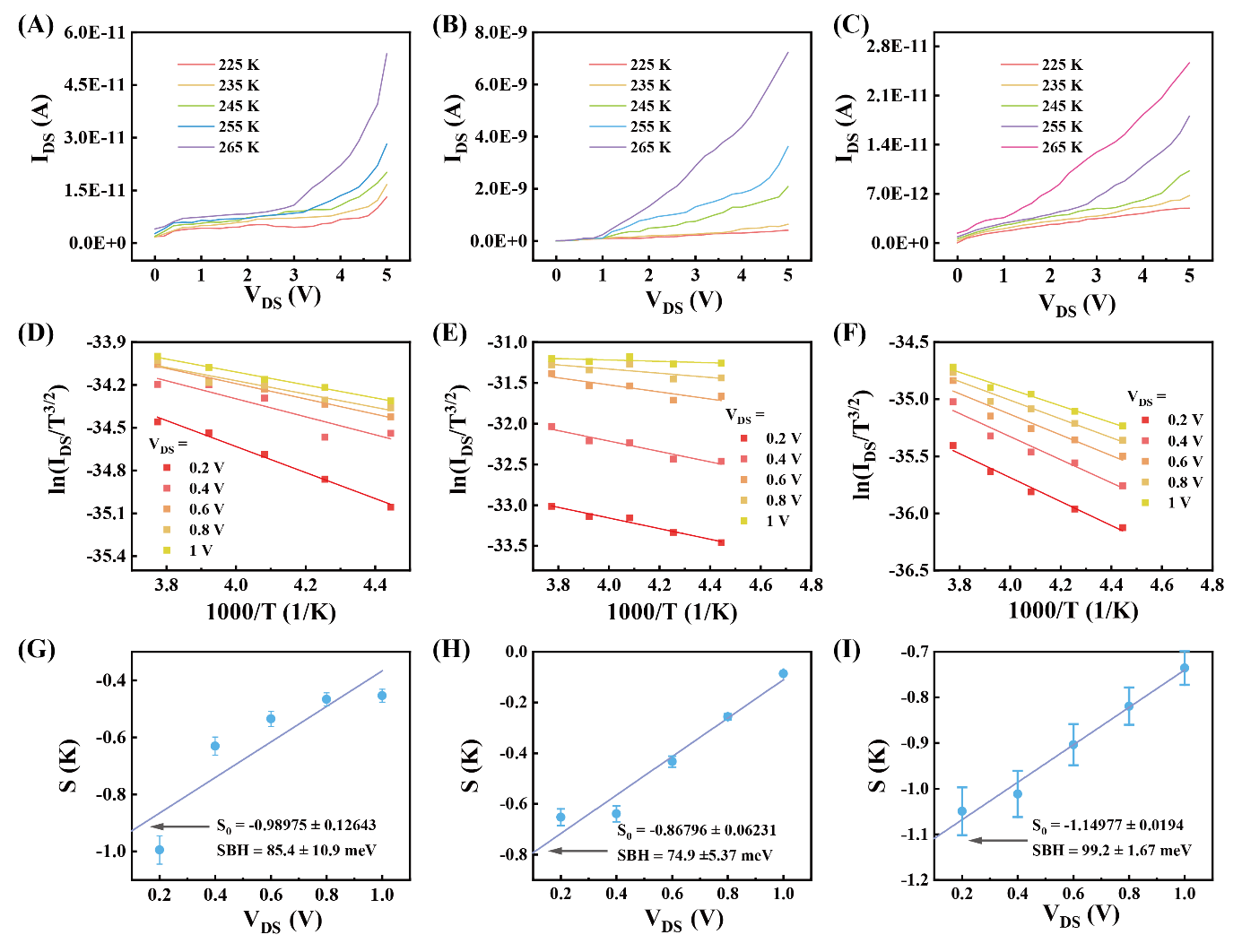


**Figure S5.** (A-C) Output curves at different temperatures from E1 (A), E2 (B), and E3 (C) FET devices. (D-F) Linear fits of the Arrehenius plot, ln(*I_DS_/T*^3/2^) vs 1000/*T* for E1 (D), E2 (E), and E3 (F) FET devices. (G-I) Slopes extracted from Figure S5 D-F as a function of *V_DS_*.

Schottky barrier height (SBH) was calculated with temperature-dependent outputs by equation:^[1]^

$I_{DS}=AA_{2D}^{*}T^{3/2}exp\left[ -\frac{q}{k_{B}T}\left( \Phi_{B}-\frac{V_{DS}}{n} \right) \right]$ (1)

Where *A* is the junction contact area, *A** is the Richardson's constant of 2D equivalent, *T* is the Kelvin temperature, *q* is the magnitude of an electron charge, *Φ_B_* is the Schottky barrier height, *k_B_* is the Boltzmann's constant, *n* is the ideal factor, and *V_DS_* is the source-drain voltage. The output curves were measured at different temperatures (**Figure S5** **A-C**). According to Equation 1, SBH can be determined by plotting ln(*I_DS_*/*T*^3/2^) vs 1000/*T* in an Arrhenius plot (**Figure S5D-F**). The slope values extracted from the curves in **Figure S5D-F**were used to obtain the intercept S_0_ on the vertical axis (**Figure S5G-I**). Based on the equation *S_0_* = -*qΦ_B_*/1000*k_B_*, the SBH was calculated.


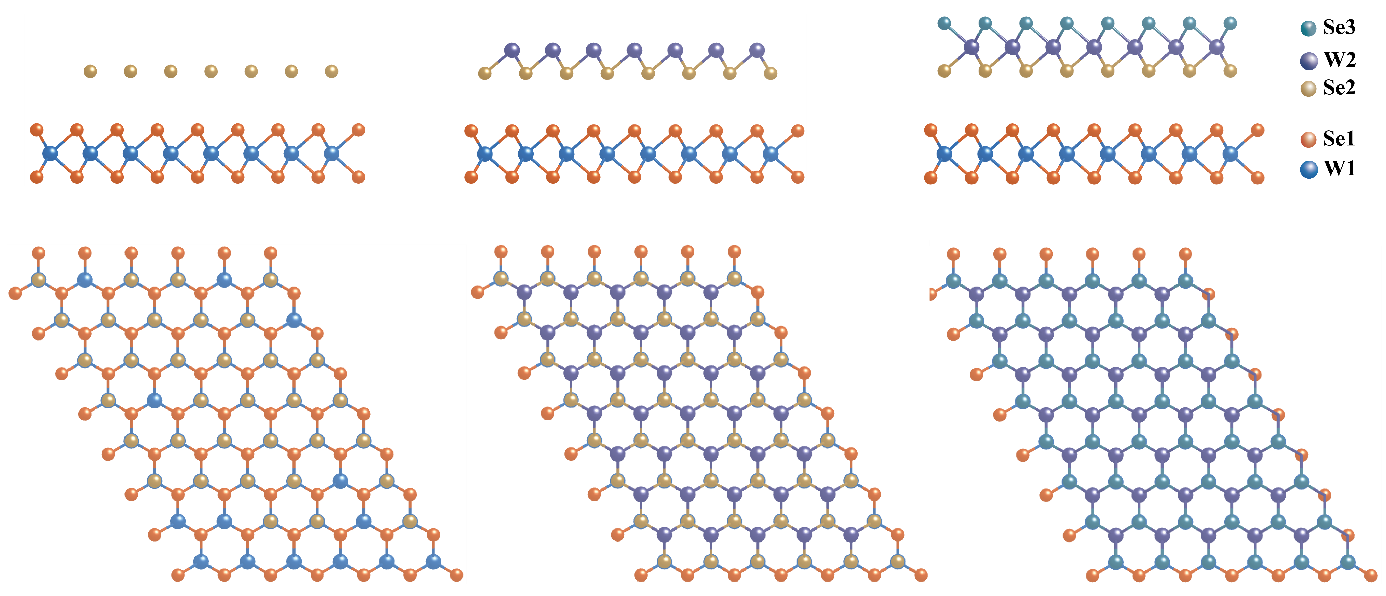


**Figure S6.** Schematic diagram of three types of intrinsic screening layers in WSe_2_.


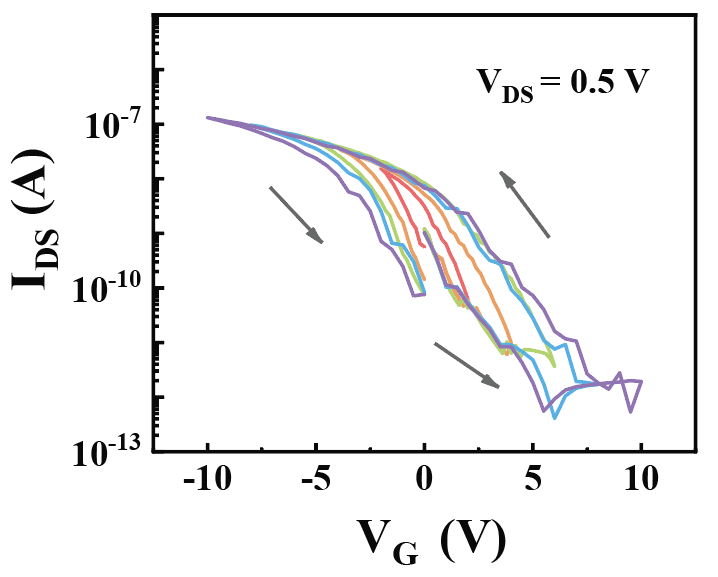


**Figure S7.** Hysteresis of transfer characteristics with different gate voltage ranges.


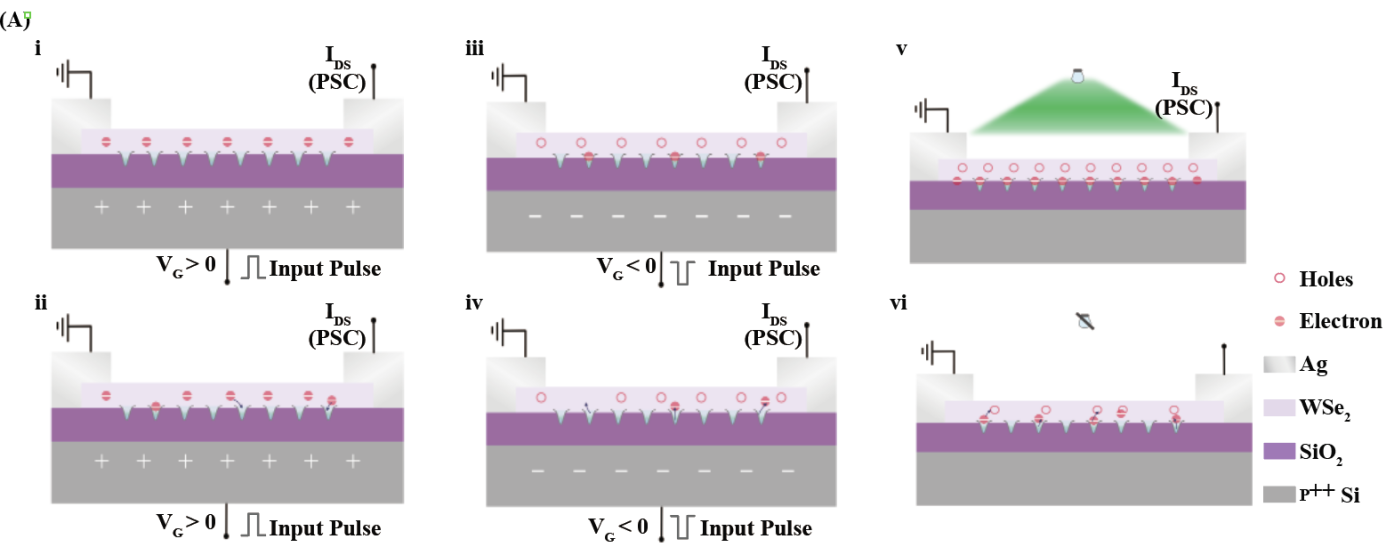


**Figure S8.** Schematic of the microscopic mechanism of charge trapping/de-trapping with positive pulses of gate voltages (i and ii), negative pulses of gate voltages (iii and iv), optical irradiation (v), and removing optical (vi), respectively.


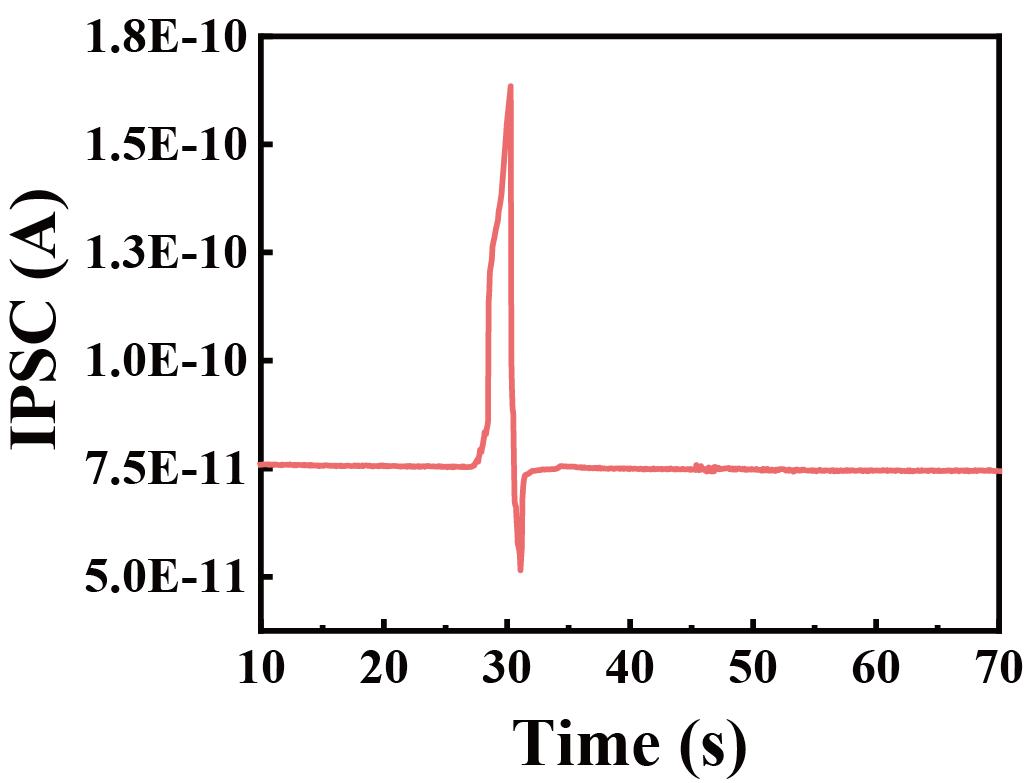


**Figure S9.** Negative pulses induce IPSC (*V* = -2 V *W* = 0.3 s).


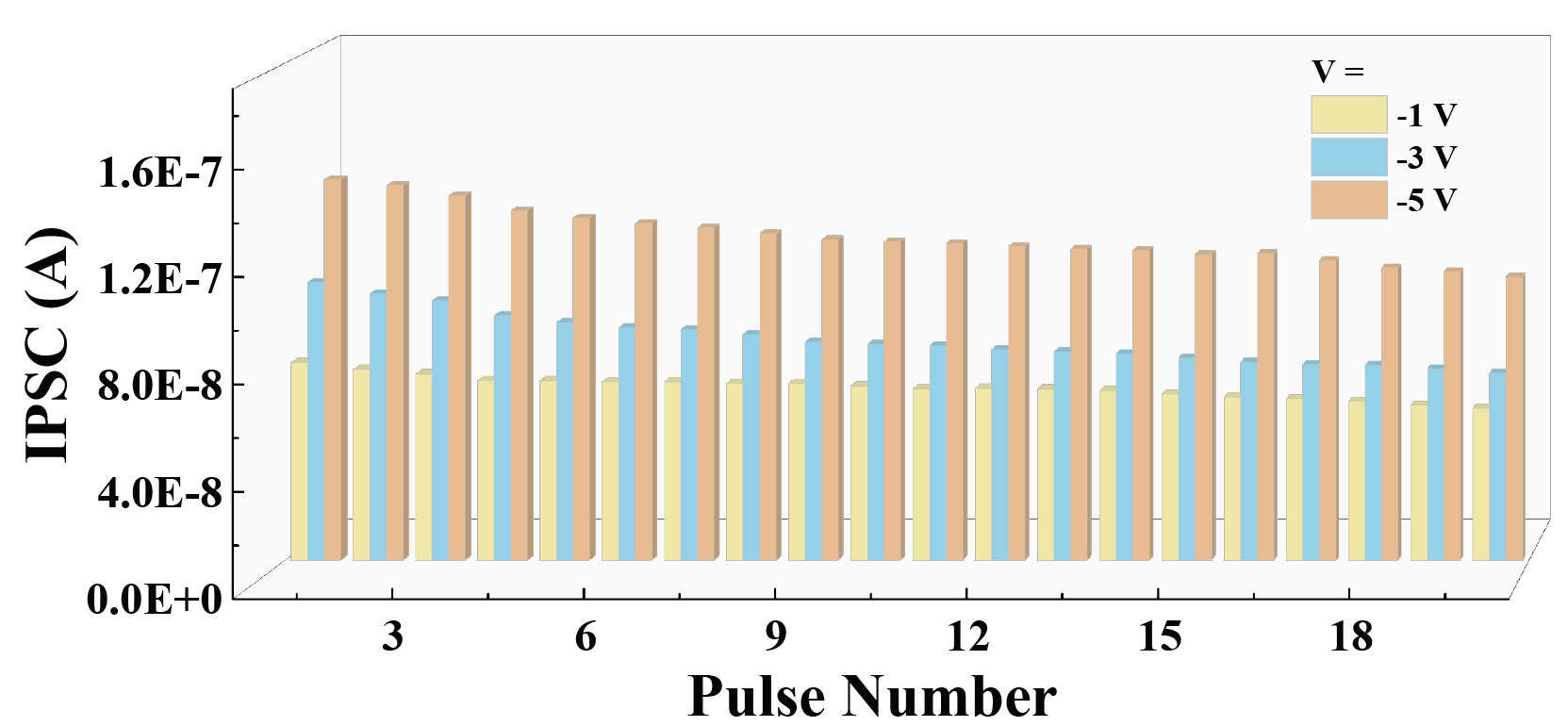


**Figure S10.** IPSC caused by voltage pulse amplitude of -1V/-3V/-5V respectively (*W* = 0.8 s *Δt* = 0.5 s).


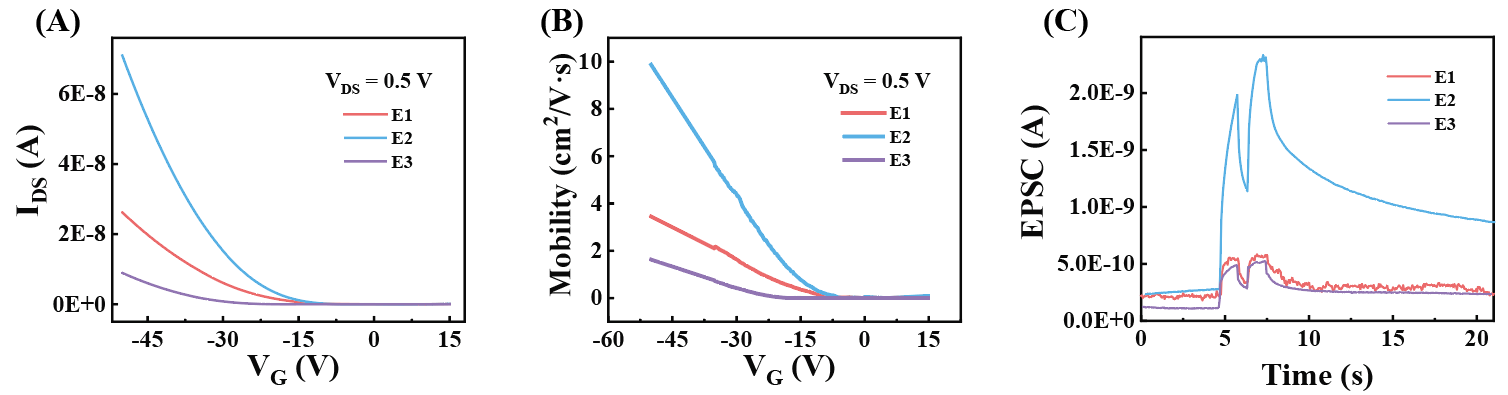


**Figure S11.** Transfer curves (A), carrier mobilities(B), and PPF (C) induced by optical pulses from E1 to E3 FETs.

The anisotropic carrier mobility from six-terminal WSe_2_ FETs was presented in **Figure S11**, where carrier mobility from E2 FET is larger than E1 and E3 FETs. when identical gate pulses or optical pulses were applied (**Figures 3B** and **4A**), the larger mobility from E2 FET induced a faster movement of carriers, leading to a higher EPSC response. While the smaller mobility from E1 and E3 FETs induced a slower movement of carriers, resulting in a smaller EPSCs responses from E1 and E3 FETs. Thus, the varying EPSC responses were presented (**Figures 3B** and **4A**). Besides, the faster movement of carriers could cause the larger number of captured electrons in defects for the larger EPSCs in E2 FET, resulting in a slower release of captured electrons in defects and a higher output currents compared to E1 and E3 FETs after removing the first pulse (**Figures 3B** and **4A**). When the second pulse was applied, the larger current would be presented based on the larger mobility and a higher basic current of E2 FET (**Figure S11**), originated from the incompletely released electrons in defects. Thus, the larger PPF behavior was shown in E2 FET compared with those from E1 and E3 FETs (**Figures 3C** and **4C**), giving the varying PPF responses.


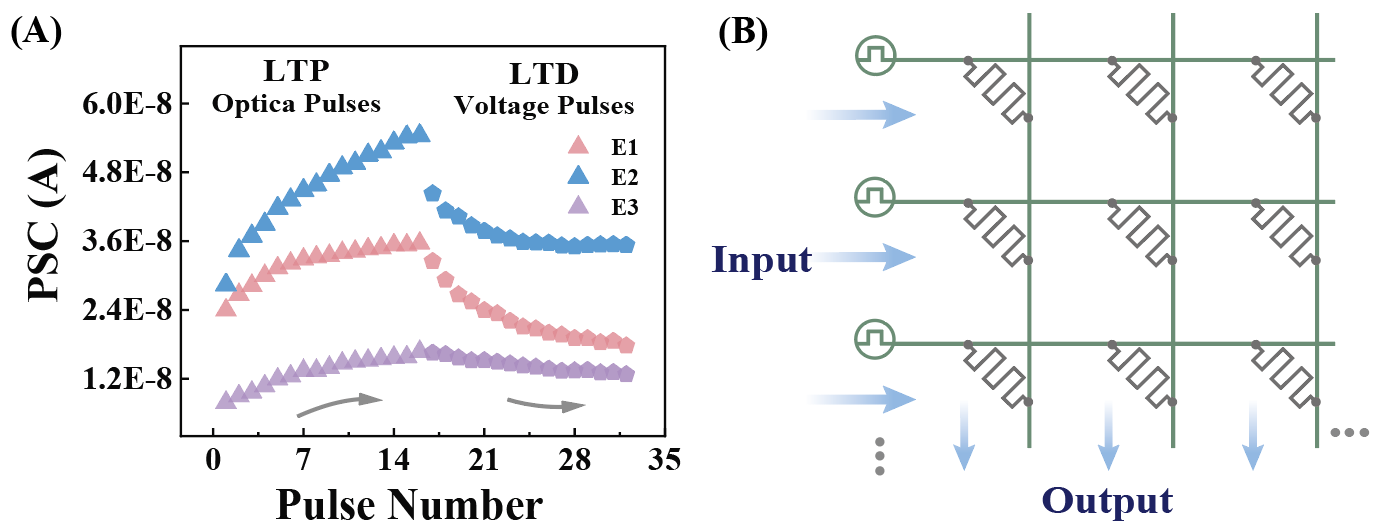


**Figure S12.** (A) Optical signal excitation LTP and voltage signal excitation LTD in artificial axonal devices (*P* = 7.28 mW/cm^2^, *W* = 0.5 s, *Δt* = 0.5 s, *V* = 0.5 V *V_DS_* = 0.5 V). (B) Crossover array structure.


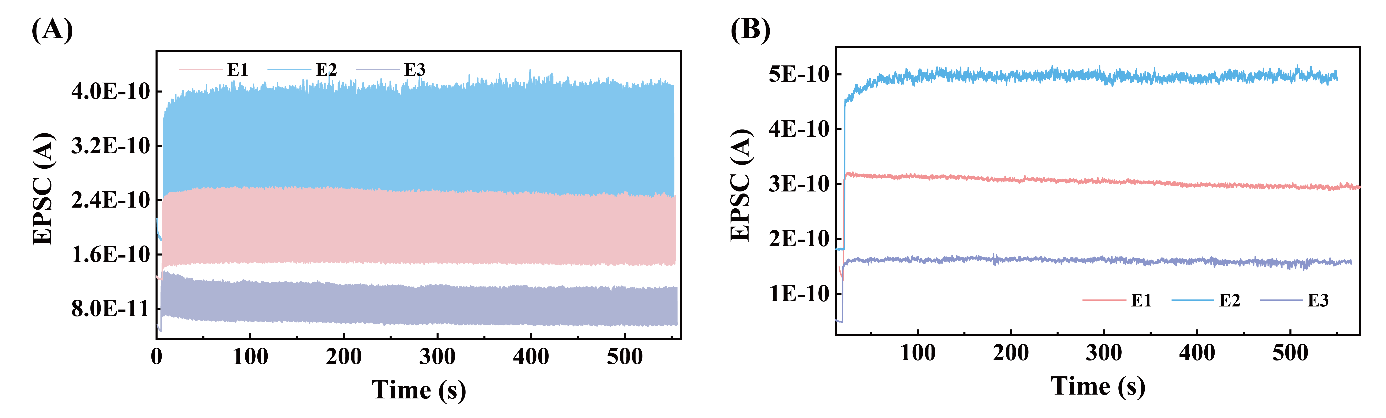


**Figure S13.** Stability tests of the multi-terminal artificial axon device. (A) Dependence of EPSC from three channels (E1, E2, E3) of the device on 900 repeated optical pulses (P = 4.1 mW/cm^2^, W= 0.3 s, Δt = 0.3 s). (B) Dependence of EPSC from three channels (E1, E2, E3) of the device on illumination time with optical power density high to 15 mW/cm^2^.

Variations and stability of multi-terminal artificial axon devices were evaluated under 900 pulses and continuous stimulation. First, 900 repeated optical pulses were applied to three anisotropic channels (**Figure S13A**). The attenuation of EPSC peaks were 3.6%, 0%, and 16.8% for E1, E2, and E3, respectively, demonstrating good cycling endurance. Second, continuous stimulation was performed with optical power density high to 15 mW/cm^2^ for 560 s (**Figure S13B**), the attenuation of EPSC for the channels were 6.4%, 0.5, and 3.2% for E1, E2, and E3, respectively, indicating a good anti-attenuation performance.


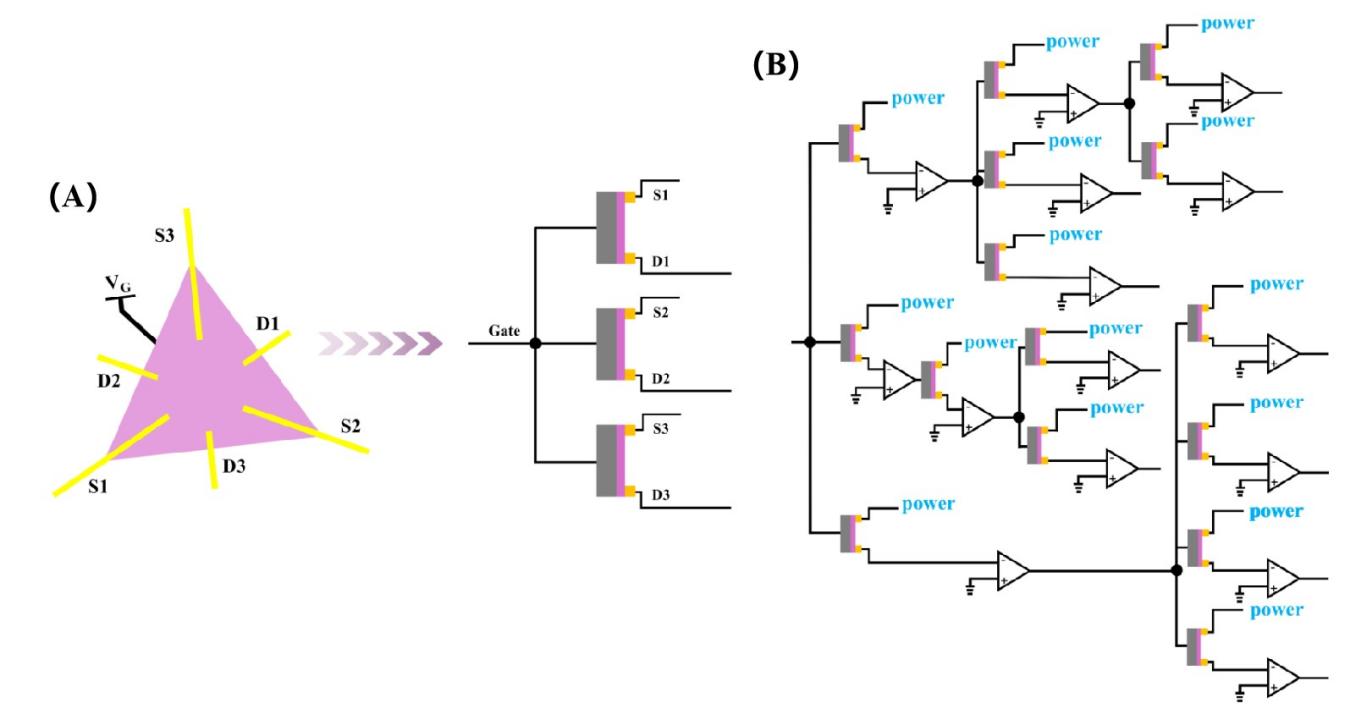


**Figure S14.** Multi-level bifurcated memristive network based on multi-terminal WSe_2_ devices. (A) Equivalent circuit of a six-terminal WSe_2_ device. (B) Schematic of the network architecture. Black dots represent electrical nodes interconnected in a tree-like manner to form a multi-path, hierarchical signal transmission structure.

A multi‑level memristive network was proposed based on multi-terminal optoelectronic WSe_2_ devices (**Figure S14**). In terms of circuit architecture, the six-terminal WSe_2_ devices could be functional as 3 transistors with sharing the same base of gate voltage (**Figure S14A**). The output of a transistor could be used as the gate voltage (base) of next transistor after being amplified and voltage conversed by a trans-impedance amplifier (**Figure S14B**). Thus a multi‑level bifurcated memristive network could be achieved by integrated multi-device circuits and their function. Due to the retention characteristics of the WSe_2_ devices, the written conductance states would be accumulated at the corresponding nodes and propagated along the network to the next level, endowing the whole system with history-dependent temporal weighting behavior.

**References**

1. Chen JR, Odenthal PM, Swartz AG, et al., “Control of Schottky Barriers in Single Layer MoS_2_ Transistors with Ferromagnetic Contacts,” *Nano Letters* (2013), 13(7):3106-3110. https://doi.org/10.1021/nl4010157
